# Preoperative evaluation of the segmental artery of left upper lobe by thin-section CT and 3d-CTA

**DOI:** 10.1007/s11604-024-01670-9

**Published:** 2024-10-09

**Authors:** Makiko Murota, Takashi Norikane, Mariko Ishimura, Yuka Yamamoto, Katsuya Mitamura, Yasukage Takami, Yuri Manabe, Katashi Satoh, Naoya Yokota, Yoshihiro Nishiyama

**Affiliations:** 1 Department of Radiology, Faculty of Medicine, Kagawa University, 1750-1 Ikenobe, Miki-cho, Kita-gun, Kagawa, 761-0793 Japan; 2 Department of Radiology, Diagnostic Imaging Center, Utazu Hospital, Utazu-cho, Kagawa, Japan; 3 Department of General Thoracic Surgery, Faculty of Medicine, Kagawa University, Kagawa, Japan

**Keywords:** Pulmonary arteries, Computed tomography angiography, Anatomy, Lung cancer

## Abstract

**Purpose:**

Understanding pulmonary artery (PA) branches and their variations is crucial for successful lung resection. We aimed to evaluate the segmental PA branching pattern of the left upper lobe (LUL) using thin-section computed tomography (TSCT) images and 3D-CT angiography (3D-CTA).

**Materials and methods:**

This study included 108 patients who underwent CTA and left upper lobectomy. The segmental PA branching pattern of the LUL was meticulously identified by two thoracic radiologists using 3D-CTA and TSCT images. The lingular artery branches from the left PA (LPA) were classified into mediastinal type (pars mediastinalis: PM), interlobar type (pars interlobaris: PI), and PI originating from the lower portion (PI’), specifically from A8. The intraoperative findings of the PA branches of the LUL were compared with the preoperatively obtained 3D-CTA and TSCT images in each patient’s case.

**Results:**

The median (range) number of LPA branches of the LUL was 4.36 (3–8). The most common number of A1 + 2 branches was two, seen in 34 cases (31.5%). One or more branches of A1 + 2c directly originating from the LPA were found in 63 cases (58.3%). The number of branches of A3 was single in 85 cases and the most frequent (78.7%). Instances where one or more branches of A3a directly originated from the LPA were found in seven cases (6.5%). A1 + 2 and A3 origins were separate and independent in 40 cases (37.0%). As the branching pattern of the lingular artery, PI/PI’ was most frequent (61.1%). PI´ was observed in 26 cases (24.1%). Inter-observer agreement for A1 + 2, A1 + 2c, A3, A3a, and lingular artery branching patterns was moderate to substantial (κ = 0.53–0.72). Preoperative 3D-CTA and TSCT images identified 99.8% of LPA branches compared to intraoperative findings, except one.

**Conclusion:**

The segmental PA branching pattern of the LUL can be evaluated using TSCT and 3D-CTA images, providing precise preoperative information.

## Introduction

Knowledge of pulmonary artery (PA) branching patterns is crucial for thoracic surgeons due to potential bleeding complications during pulmonary resection. Anatomic PA branching pattern variations in the left upper lobe are more common than the right, complicating lung resection, particularly when interlobar fissure separation is incomplete. Hence, preoperative identification of PA branches in the left upper lobe is essential for ensuring patient safety and facilitating lung resection [1–3].

Recent studies have demonstrated the utility of segmentectomy for early-stage ground-glass opacity predominant lung cancer and a diameter of ≤ 2 cm, leading to an increased adoption of this approach [4, 5]. This underscores the need for detailed information on PA branching patterns at segmental and peripheral levels. Although preoperative evaluation using three-dimensional computed tomography (CT) angiography (3D-CTA) has shown utility [6–12], investigations involving a substantial number of cases of segmental PA branching patterns of the left upper lobe using thin-section CT images and 3D-CTA remain limited. Moreover, there is still a lack of substantial comparisons between preoperative imaging and intraoperative findings to support its utility in the left upper lobe [12, 13].

This study aims to evaluate segmental PA branching patterns of the left upper lobe using thin-section CT images and 3D-CTA in order to provide insights into the understanding of PA branches and their variations essential for successful anatomical lung resections.

## Materials and methods

### Patients

**Fig. 1:**
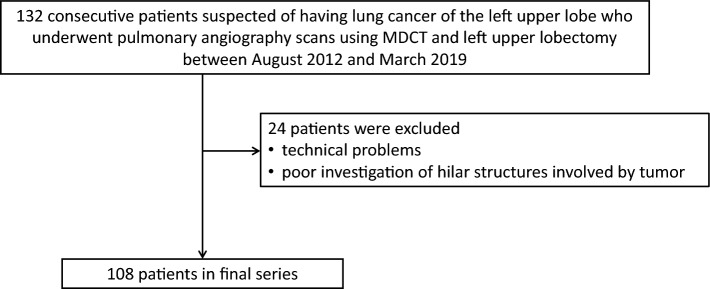
Study flowchart

The Ethics Committee of our hospital approved this retrospective study and waived the need for obtaining individual patient consent. A total of 132 consecutive patients with suspected left upper lobe lung cancer who had undergone pulmonary angiography using multidetector row CT (MDCT) and left upper lobectomy between August 2012 and March 2019 were retrospectively reviewed. After excluding 24 patients who had inadequate investigation of tumor-involved hilar structures and technical problems, the final cohort comprised 108 patients (59 men and 49 women; mean age, 69.0 years; age range, 14–85 years) (Fig. 1). Some data from 99 of these patients were previously utilized in another study [12].

### Contrast-enhanced MDCT

Both 64-slice MDCT (Aquilion 64, Toshiba Medical Systems, Tokyo, Japan) and 256-slice MDCT (Brilliance iCT, Philips Healthcare, Cleveland, OH, USA) scanners were used. The technical parameters for the 64- vs. 256-slice MDCT, respectively, were as follows: a detector row configuration of 0.5 mm vs. 0.625 mm, a pitch of 53 vs. 106 (detector pitch of 0.83 vs. 0.83), a reconstruction increment of 0.4 mm vs. 0.5 mm, and a section thickness of 0.5 mm vs. 0.67 mm. An x-ray tube voltage of 120 kV and an automatic exposure control for tube current were used in all examinations. The examinations were performed with the patient in the supine position during a single breath hold at end-inspiration.

A dual-head power injector (Dual Shot GX, Nemoto Kyorindo, Tokyo, Japan) was used for all patients for the bolus administration of the contrast material iohexol (Omnipaque 350, GE Healthcare Pharma, Tokyo, Japan) or iopamidol (Iopamiron 300, Bayer Yakuhin, Osaka, Japan) via a cubital vein. In patients weighing ≥ 55 kg, 100 mL of iohexol (350 mg iodine/mL) was injected at a rate of 3.3 mL/s, and scanning was performed 18 s afterwards. In patients with a body weight of 44–55 kg, 85 mL of iohexol (350 mg iodine/mL) was injected at a rate of 3.3 mL/s, and scanning was performed 15 s later. In patients weighing < 44 kg, 85 mL of iopamidol (300 mg iodine/mL) was injected at a rate of 3.3 mL/s, and scanning was performed 15 s later.

Another protocol for PA and pulmonary vein (PV) separation images was determined from the time-density curve using a test bolus dose. The injection rate was 4 mL/s, with a 20-mL test bolus injected prior to the main injection. The test injection determined the adequate timing for the PA/PV scan. The PA/PV scan was performed with 50 mL of iohexol (350 mg iodine/mL). The saline chaser was 40 mL, and the injection rate was 4 mL/s. These two protocols were comparable to investigating the PA branching pattern in detail. The volume data obtained from the arterial phase were transferred to a workstation (Zio STATION, Ziosoft, Tokyo, Japan), where the data were converted to a 3D-CTA format using the volume-rendering technique.

### Image analysis

Thin-section transverse images were reviewed at a width of 1600 HU and level -200 HU window settings with paging on a viewer (EV insite, PSP Corporation, Tokyo, Japan). The 3D-CTA images were interpreted by rotating the same viewer. The window, level, and opacity of the volumes were subjectively selected to optimize PA visualization. In the present study, the number and origin of PA branches in the left upper lobe were meticulously identified using 3D-CTA and thin-section images on the same viewer. These images were reviewed with an interval of several days between interpretations.

Two board-certified thoracic radiologists, with 12 and 22 years of experience, respectively, independently reviewed each CT image. In cases of discrepancy over branching, the images were re-evaluated with both 3D-CTA and thin-section images until a consensus was reached to avoid interobserver variability. The intraoperative findings of the PA branches of the left upper lobe were compared with the preoperatively obtained 3D-CTA and thin-section images in each patient’s case.

The nomenclature used to describe the segmental PA is that of Yamashita [14]. The branches to the left upper lobe arise from the anterior, posterosuperior, and interlobar portions of the vessel: A1 + 2, A3, A4, and A5. The lingular artery (A4 and A5) originates from the interlobar portion (pars interlobaris [PI]) of the left PA (LPA) and may arise from the anterior portion of the mediastinal part of the left arterial trunk (pars mediastinalis [PM]). Moreover, the lingular arteries of PI may sometimes originate from the lower portion, from A8 or the common trunk of A8 and A9 [7]. In the present study, the lingular arteries are identified separately as PI and PI originated from the lower portion (from A8 or the common trunk of A8 and A9, denoted as PI’) [12, 15].

### Statistical analysis

Statistical analyses were performed in SPSS version 26 (IBM Corp., Armonk, NY). Inter-observer agreement regarding the PA branching pattern in the left upper lobe was analyzed by calculating Cohen’s kappa coefficient before reaching a consensus with the thoracic radiologist who reviewed the images. The inter-observer agreement was classified as follows: excellent (κ = 0.81–1.00), substantial (κ = 0.61–0.80), moderate (κ = 0.41–0.60), fair (κ = 0.21–0.40), and poor (κ = 0–0.20).

## Results

**Fig. 2:**
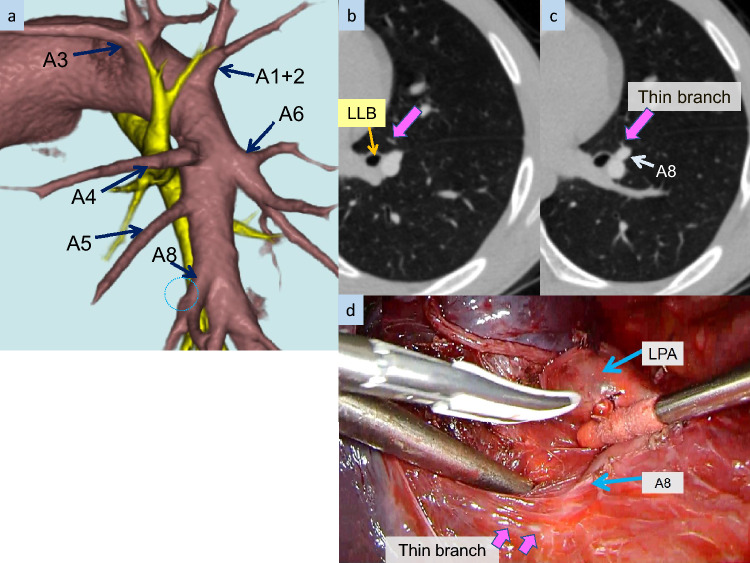
3D-CTA, thin-section CT, and intraoperative images in a male teenager suspected of having lung cancer of the left upper lobe. **a–c** The thin branch of the PA supplying the lingular segment branching off from A8 is depicted on thin-section CT images, although not visualized on 3D-CTA images. **d** The thin branch was identified intraoperatively

The median (range) number of branches of the PA of the left upper lobe, upper division segment, and lingular segment was 4.36 (3–8), 2.62 (1–3), and 1.74 (1–4), respectively. Tables 1, 2, and 3 show the branching patterns of the PA of the left upper lobe according to the 3D-CTA and thin-section images. The number of branches of A1 + 2 was two in 34 cases and was most frequent at 31.5% (34/108). However, splits (24.1%), meaning separate branching of subsegmental arteries from both A1 + 2 and A3, or even four branches (12.0%), were observed. For the branching pattern of A1 + 2c, two branches were found in 25 cases (23.1%), one or more branches of A1 + 2c directly originated from the LPA in 63 cases (58.3%), and two branches of A1 + 2c directly originated from the LPA and PI in 4 cases (3.7%). The number of branches of A3 was single in 85 cases, making it the most frequent at 85 (78.7%). The relationship between A1 + 2 and A3 origins was that they originated separately and independently in 40 cases (37.0%). For A3a, two branches were found in 8 cases (7.4%), one or more branches of A3a directly originated from the LPA of the interlobar portion in seven cases (6.5%), and a branch of A3a directly originated from PI in 6 cases (5.6%). As for the branching pattern of the lingular segment, PI (including PI’) was the most frequent at 61.1% (66/108). PI’ was observed in 26 cases (24.1%) (Fig. 2). “Other” was a case with branches originating from A7 in addition to the PI.

**Table 1:** The branching pattern of A1 + 2 (n = 108)

	Number of patients (%) (n = 108)		
Single stem	12 (11.1%)		
		A1 + 2abc	12 (11.1%)
Two stems	34 (31.5%)		
		A1 + 2ab, A1 + 2c	17 (15.7%)
		A1 + 2bc, A1 + 2a	16 (14.8%)
		A1 + 2ca, A1 + 2b	1 (0.9%)
Three stems	23 (21.3%)		
		A1 + 2a, A1 + 2b, A1 + 2c	19 (17.6%)
		A1 + 2ab, two branches of A1 + 2c	4 (3.7%)
Four stems	13 (12.0%)		
		A1 + 2a, A1 + 2b, two branches of A1 + 2c	10 (9.3%)
		Two branches of A1 + 2a, A1 + 2b, A1 + 2c	1 (0.9%)
		Others	2 (1.9%)
Split	26 (24.1%)	Split	26 (24.1%)

Split: subsegmental artery originates separately from both A1 + 2 and A3

**Table 2:** The branching pattern of A3

	Number of patients (%) (n = 108)		
Single stem	85 (78.7%)		
		A3a, A3b, A3c	85 (78.7%)
Two stems	12 (11.1%)		
		A3ab, A3c	1 (0.9%)
		A3bc, A3a	6 (5.6%)
		A3ca, A3b	5 (4.6%)
Split	11 (10.2%)	Split	11 (10.2%)

Split: subsegmental artery originates separately from both A1 + 2 and A3

**Table 3:** The branching pattern of the lingular arteries

	Number of patients (%) (n = 108)		
Lingular arteries			
PM	5 (4.6%)	PM	5 (4.6%)
PI (including PI’)	66 (61.1%)		
		PI	48 (44.4%)
		PI, PI’	14 (13.0%)
		PI’	4 (3.7%)
PM, PI (including PI’)	36 (33.3%)		
		PM, PI	28 (25.9%)
		PM, PI’	3 (2.8%)
		PM, PI, PI’	5 (4.6%)
Other	1 (1.0%)	Other	1 (1.0%)

*PM* pars mediastinalis, *PI* pars interlobaris, *PI'*’ PI originating from A8 or the common trunk of A8 and A9

The inter-observer agreement for the branching pattern of A1 + 2, A1 + 2c, A3, A3a, and the lingular segmental artery, as well as the number of PI’, was moderate (κ = 0.59), substantial (κ = 0.69), moderate (κ = 0.56), moderate (κ = 0.53), substantial (κ = 0.72), and moderate (κ = 0.53), respectively.

Preoperative 3D-CTA and thin-section CT images identified 99.8% of LPA branches compared to the intraoperative findings, with only one exception.

## Discussion

Recent studies have demonstrated the utility of segmentectomy for early-stage ground-glass opacity predominant lung cancer and a diameter of ≤ 2 cm, leading to an increased adoption of this approach [4, 5]. This underscores the importance of detailed information on PA branching patterns at the segmental and subsegmental levels. In this study, the PA branching pattern of the left upper lobe before lobectomy was evaluated using 3D-CTA images and thin-section images and compared with intraoperative findings. The number of branches of the left upper lobe PA ranged from 3 to 8, consistent with previous investigations [16, 17].

Similar to Yamashita’s study on the bronchovascular anatomy of 165 specimens [14], it was observed that A1 + 2 often has multiple stems (Yamashita: 89.1%; present study: 88.9%), while A3 commonly arises as a single stem (Yamashita: 77.0%; present study: 78.7%). A1 + 2c is known to arise directly from the PA, as observed in 54.6% in Yamashita’s study and 58.3% in the present study. When the branching pattern of A3 is other than a single stem, A3a may unexpectedly originate from various locations, including branching from the PI and directly from the LPA.

Cases with only PM branching of the lingular artery are infrequent, as demonstrated in the present study (4.6%). Furthermore, the lingular artery often branches from below the bifurcated A8, termed PI’, in approximately a quarter of cases. The presence of this branching is considered important not only during upper or lower lobe resections but also during segmental resections [12, 15]. However, PI’ branches off from the caudal peripheral side of the LPA, making it frequently thinner than PI. Since the thin branches are not depicted by 3D-CTA and are seen only on thin-section CT, the presence of PI’ should be known and carefully checked for on thin-section CT (Fig. 2).

Several reports have demonstrated the depiction of PA branches using 3D-CTA compared with intraoperative findings. For instance, previous studies have demonstrated that 95–99.7% of PA branches can be identified using 3D-CTA and thin-section CT images [6, 9–11]. However, some fine branches cannot be depicted using 3D-CTA alone, making confirmation with thin-section CT essential. The use of contrast agents allows for creating high-quality 3D-CTA images of PAs. While 3D-CTA is crucial for surgeons to visualize PA branching patterns during preoperative simulation, it is necessary to distinguish, in detail, the PA branching patterns at the subsegmental level. For instance, in cases like A1 + 2c or A3a, two branches may exist or originate from the PI, making it essential to assess PA branching in relation to subsegmental and more peripheral bronchial branching patterns. Therefore, it is indispensable to use both 3D-CTA and thin-section CT images for such assessments. This approach also improves interobserver agreement. In this study, we used both 3D-CTA and thin-section CT for evaluation; however, we did not compare the findings of individual methods, namely thin-section CT or 3D-CTA, with intraoperative findings nor did we assess interobserver agreement. This is due to the aforementioned reasons, as well as the fact that the depiction of branches can easily change depending on the rendering method in 3D imaging. Additionally, with increased reading experience, it becomes possible to recognize a potential branch in a subtle elevation that might initially appear as an absence PA branching. Therefore, we believe it is essential to use both imaging modalities for thorough evaluation.

In this study, we examined the concordance rate for PA branching patterns of the left upper lobe, obtaining substantial and moderate results from two radiologists. Discrepancies were primarily due to differences in how the segments were delineated, with some cases where less experienced readers missed thin arteries. It is crucial to consider the possibility of classification differences due to evaluator variability. Our study is the first to investigate interobserver agreement on PA branching using 3D-CTA and thin-section CT. This highlights the need to explore methods to improve preoperative evaluation consistency among clinicians—such as thoracic surgeons and radiologists—in future studies, ensuring that even less experienced physicians can better recognize thin branches.

Similar to reports comparing 3D-CTA and thin-section CT with intraoperative findings on the PA branching patterns of the anatomically variable right upper lobe, where 99.7% of PA branches were identified [11], there have been no reports with a substantial number of cases for the similarly variable left upper lobe. This study is the first to compare 3D-CTA and thin-section CT with intraoperative findings in the left upper lobe and achieved a 99.8% detection rate of PA branches. On the left side, there were concerns about artifacts due to cardiac motion potentially obscuring fine branches of the lingular segment. However, the comparison with intraoperative findings demonstrated depiction capabilities equivalent to those on the right upper lobe, alleviating these concerns. This study shows that 3D-CTA is sufficient for preoperative evaluation even in the left upper lobe, demonstrating comparable efficacy to intraoperative findings.

The present study had some limitations. First, its retrospective nature introduced a potential selection bias. Second, both 64-slice and 256-slice MDCT scanners were utilized. We analyzed the PA branching pattern of the left upper lobe using 3D-CTA and thin-section CT images with two different CTA protocols. However, we believe that these differences did not impact the results, as the CT images were adequate for investigating the PA branching patterns. Finally, although we compared 3D-CTA and thin-section CT with intraoperative findings, this study focused solely on the branching patterns from the LPA in left upper lobe resections. However, the high concordance rate observed with intraoperative findings for the LPA suggests that the branching patterns identified by CT are likely to reflect the actual anatomy with high accuracy. To further advance segmentectomy and subsegmentectomy techniques, future research should investigate the more peripheral PA branches.

In conclusion, 3D-CTA and thin-section images provided precise preoperative information regarding the subsegmental PA branching pattern in the left upper lobe. Despite initial concerns, imaging of the left upper lobe was comparable to intraoperative findings seen in a previous study on the right upper lobe. It is important to utilize thin-section CT alongside 3D-CTA for accurate identification of smaller branches like PI’. These findings support the utilization of 3D-CTA and thin-section CT for preoperative evaluations in left upper lobe surgeries, contributing to the safety and ease of lobar or segmental left upper lobe resection.
